# Supplementary material for: Modeling glioblastoma heterogeneity as a dynamic network of cell states
Source: Mol Syst Biol. 2021 Sep 16;17(9):e10105. doi: 10.15252/msb.202010105 (PMC8444284; doi:10.15252/msb.202010105)
Supplement: Supplementary file 5 — Source Data for Figure 3 [file MSB-17-e10105-s001.zip › Figure3A_sourcedata/GSEA_3065/hallmarks_state6.GseaPreranked.1623416308702/HALLMARK_ADIPOGENESIS.html]

Details for gene set HALLMARK\_ADIPOGENESIS[GSEA]

|  || Dataset | state6 |
| Phenotype | NoPhenotypeAvailable |
| Upregulated in class | na\_pos |
| GeneSet | HALLMARK\_ADIPOGENESIS |
| Enrichment Score (ES) | 0.21549962 |
| Normalized Enrichment Score (NES) | 0.96729934 |
| Nominal p-value | 0.5804749 |
| FDR q-value | 0.55863285 |
| FWER p-Value | 1.0 |
Table: GSEA Results Summary

  

Fig 1: Enrichment plot: HALLMARK\_ADIPOGENESIS      
 Profile of the Running ES Score & Positions of GeneSet Members on the Rank Ordered List

  

| PROBE | GENE SYMBOL | GENE\_TITLE | RANK IN GENE LIST | RANK METRIC SCORE | RUNNING ES | CORE ENRICHMENT || 1 | GADD45A |  |  | 2 | 0.419 | 0.0401 | Yes |
| 2 | UQCR11 |  |  | 71 | 0.230 | 0.0544 | Yes |
| 3 | VEGFB |  |  | 73 | 0.229 | 0.0763 | Yes |
| 4 | COX8A |  |  | 117 | 0.205 | 0.0910 | Yes |
| 5 | SUCLG1 |  |  | 221 | 0.169 | 0.0954 | Yes |
| 6 | UQCR10 |  |  | 257 | 0.161 | 0.1068 | Yes |
| 7 | GPX4 |  |  | 365 | 0.141 | 0.1080 | Yes |
| 8 | CMBL |  |  | 402 | 0.136 | 0.1169 | Yes |
| 9 | SLC25A1 |  |  | 437 | 0.130 | 0.1255 | Yes |
| 10 | CMPK1 |  |  | 462 | 0.127 | 0.1349 | Yes |
| 11 | RAB34 |  |  | 467 | 0.127 | 0.1467 | Yes |
| 12 | NDUFA5 |  |  | 492 | 0.124 | 0.1559 | Yes |
| 13 | AGPAT3 |  |  | 496 | 0.124 | 0.1674 | Yes |
| 14 | UQCRC1 |  |  | 613 | 0.113 | 0.1649 | Yes |
| 15 | SLC27A1 |  |  | 664 | 0.109 | 0.1696 | Yes |
| 16 | RIOK3 |  |  | 687 | 0.107 | 0.1773 | Yes |
| 17 | TOB1 |  |  | 746 | 0.102 | 0.1805 | Yes |
| 18 | COX7B |  |  | 800 | 0.098 | 0.1838 | Yes |
| 19 | MGST3 |  |  | 824 | 0.097 | 0.1904 | Yes |
| 20 | ATP5PO |  |  | 856 | 0.094 | 0.1959 | Yes |
| 21 | SLC25A10 |  |  | 862 | 0.094 | 0.2043 | Yes |
| 22 | ITSN1 |  |  | 895 | 0.091 | 0.2094 | Yes |
| 23 | CHCHD10 |  |  | 918 | 0.090 | 0.2155 | Yes |
| 24 | SCP2 |  |  | 1067 | 0.082 | 0.2063 | No |
| 25 | UQCRQ |  |  | 1209 | 0.075 | 0.1972 | No |
| 26 | PDCD4 |  |  | 1248 | 0.074 | 0.1999 | No |
| 27 | UCK1 |  |  | 1250 | 0.074 | 0.2069 | No |
| 28 | PGM1 |  |  | 1302 | 0.071 | 0.2078 | No |
| 29 | QDPR |  |  | 1420 | 0.067 | 0.2007 | No |
| 30 | ETFB |  |  | 1467 | 0.066 | 0.2017 | No |
| 31 | NDUFS3 |  |  | 1504 | 0.064 | 0.2037 | No |
| 32 | ACAA2 |  |  | 1540 | 0.062 | 0.2057 | No |
| 33 | UBC |  |  | 1698 | 0.057 | 0.1930 | No |
| 34 | COQ5 |  |  | 1866 | 0.052 | 0.1786 | No |
| 35 | SORBS1 |  |  | 1888 | 0.051 | 0.1811 | No |
| 36 | CAVIN1 |  |  | 1909 | 0.050 | 0.1836 | No |
| 37 | UCP2 |  |  | 2041 | 0.047 | 0.1730 | No |
| 38 | NDUFB7 |  |  | 2093 | 0.045 | 0.1714 | No |
| 39 | MRPL15 |  |  | 2130 | 0.044 | 0.1715 | No |
| 40 | PHLDB1 |  |  | 2185 | 0.042 | 0.1693 | No |
| 41 | JAGN1 |  |  | 2196 | 0.042 | 0.1721 | No |
| 42 | PTCD3 |  |  | 2224 | 0.040 | 0.1729 | No |
| 43 | MYLK |  |  | 2302 | 0.038 | 0.1677 | No |
| 44 | ARAF |  |  | 2338 | 0.038 | 0.1673 | No |
| 45 | BCL6 |  |  | 2351 | 0.037 | 0.1695 | No |
| 46 | COQ9 |  |  | 2383 | 0.036 | 0.1694 | No |
| 47 | IDH1 |  |  | 2508 | 0.033 | 0.1582 | No |
| 48 | GBE1 |  |  | 2517 | 0.033 | 0.1605 | No |
| 49 | MGLL |  |  | 2542 | 0.032 | 0.1608 | No |
| 50 | MIGA2 |  |  | 2691 | 0.029 | 0.1465 | No |
| 51 | MCCC1 |  |  | 2841 | 0.026 | 0.1317 | No |
| 52 | UBQLN1 |  |  | 2845 | 0.026 | 0.1338 | No |
| 53 | AIFM1 |  |  | 2865 | 0.025 | 0.1340 | No |
| 54 | SCARB1 |  |  | 2923 | 0.024 | 0.1297 | No |
| 55 | CCNG2 |  |  | 2949 | 0.023 | 0.1290 | No |
| 56 | GHITM |  |  | 2976 | 0.022 | 0.1282 | No |
| 57 | PPM1B |  |  | 3068 | 0.021 | 0.1196 | No |
| 58 | ANGPT1 |  |  | 3104 | 0.020 | 0.1175 | No |
| 59 | ARL4A |  |  | 3127 | 0.019 | 0.1168 | No |
| 60 | AK2 |  |  | 3141 | 0.019 | 0.1171 | No |
| 61 | ABCA1 |  |  | 3161 | 0.019 | 0.1167 | No |
| 62 | PIM3 |  |  | 3179 | 0.018 | 0.1165 | No |
| 63 | ECH1 |  |  | 3187 | 0.018 | 0.1174 | No |
| 64 | PREB |  |  | 3212 | 0.017 | 0.1163 | No |
| 65 | DECR1 |  |  | 3363 | 0.014 | 0.1003 | No |
| 66 | ACADM |  |  | 3368 | 0.014 | 0.1012 | No |
| 67 | GPD2 |  |  | 3376 | 0.014 | 0.1017 | No |
| 68 | YWHAG |  |  | 3404 | 0.013 | 0.0998 | No |
| 69 | DLAT |  |  | 3553 | 0.010 | 0.0837 | No |
| 70 | LPCAT3 |  |  | 3698 | 0.007 | 0.0677 | No |
| 71 | IDH3G |  |  | 3728 | 0.006 | 0.0649 | No |
| 72 | NDUFAB1 |  |  | 3836 | 0.004 | 0.0529 | No |
| 73 | SAMM50 |  |  | 3850 | 0.004 | 0.0518 | No |
| 74 | CS |  |  | 3864 | 0.003 | 0.0506 | No |
| 75 | SDHC |  |  | 3913 | 0.002 | 0.0452 | No |
| 76 | MTCH2 |  |  | 3966 | 0.001 | 0.0394 | No |
| 77 | MAP4K3 |  |  | 3972 | 0.001 | 0.0389 | No |
| 78 | CAT |  |  | 3983 | 0.001 | 0.0378 | No |
| 79 | ALDOA |  |  | 4006 | 0.001 | 0.0353 | No |
| 80 | ALDH2 |  |  | 4208 | -0.003 | 0.0124 | No |
| 81 | DDT |  |  | 4257 | -0.004 | 0.0072 | No |
| 82 | DRAM2 |  |  | 4267 | -0.004 | 0.0066 | No |
| 83 | NMT1 |  |  | 4270 | -0.004 | 0.0068 | No |
| 84 | PFKL |  |  | 4347 | -0.006 | -0.0014 | No |
| 85 | PHYH |  |  | 4372 | -0.006 | -0.0036 | No |
| 86 | HADH |  |  | 4431 | -0.007 | -0.0096 | No |
| 87 | PLIN2 |  |  | 4478 | -0.008 | -0.0141 | No |
| 88 | DGAT1 |  |  | 4639 | -0.011 | -0.0315 | No |
| 89 | RNF11 |  |  | 4680 | -0.012 | -0.0349 | No |
| 90 | TANK |  |  | 4755 | -0.014 | -0.0422 | No |
| 91 | DNAJC15 |  |  | 4857 | -0.016 | -0.0524 | No |
| 92 | ACLY |  |  | 4883 | -0.016 | -0.0537 | No |
| 93 | COQ3 |  |  | 5019 | -0.019 | -0.0675 | No |
| 94 | TST |  |  | 5025 | -0.019 | -0.0663 | No |
| 95 | PEMT |  |  | 5166 | -0.022 | -0.0804 | No |
| 96 | CRAT |  |  | 5200 | -0.022 | -0.0821 | No |
| 97 | ESRRA |  |  | 5209 | -0.022 | -0.0808 | No |
| 98 | STOM |  |  | 5231 | -0.023 | -0.0811 | No |
| 99 | GPAT4 |  |  | 5257 | -0.023 | -0.0817 | No |
| 100 | CPT2 |  |  | 5278 | -0.024 | -0.0818 | No |
| 101 | NKIRAS1 |  |  | 5410 | -0.027 | -0.0944 | No |
| 102 | ADIPOR2 |  |  | 5514 | -0.029 | -0.1035 | No |
| 103 | ACOX1 |  |  | 5517 | -0.029 | -0.1010 | No |
| 104 | PRDX3 |  |  | 5634 | -0.031 | -0.1114 | No |
| 105 | ECHS1 |  |  | 5643 | -0.031 | -0.1093 | No |
| 106 | IDH3A |  |  | 5673 | -0.032 | -0.1095 | No |
| 107 | TKT |  |  | 5888 | -0.036 | -0.1309 | No |
| 108 | DNAJB9 |  |  | 5974 | -0.037 | -0.1371 | No |
| 109 | ELMOD3 |  |  | 6243 | -0.043 | -0.1639 | No |
| 110 | DLD |  |  | 6247 | -0.044 | -0.1600 | No |
| 111 | CYC1 |  |  | 6383 | -0.047 | -0.1712 | No |
| 112 | DBT |  |  | 6411 | -0.047 | -0.1697 | No |
| 113 | MDH2 |  |  | 6431 | -0.048 | -0.1673 | No |
| 114 | BCL2L13 |  |  | 6645 | -0.054 | -0.1868 | No |
| 115 | FAH |  |  | 6682 | -0.055 | -0.1856 | No |
| 116 | SLC5A6 |  |  | 6734 | -0.057 | -0.1860 | No |
| 117 | SDHB |  |  | 6839 | -0.060 | -0.1923 | No |
| 118 | RMDN3 |  |  | 6992 | -0.065 | -0.2037 | No |
| 119 | HIBCH |  |  | 7012 | -0.065 | -0.1996 | No |
| 120 | ADCY6 |  |  | 7053 | -0.066 | -0.1978 | No |
| 121 | ACO2 |  |  | 7057 | -0.066 | -0.1918 | No |
| 122 | SOD1 |  |  | 7066 | -0.067 | -0.1863 | No |
| 123 | COX6A1 |  |  | 7089 | -0.067 | -0.1823 | No |
| 124 | LIFR |  |  | 7101 | -0.068 | -0.1771 | No |
| 125 | ESYT1 |  |  | 7297 | -0.074 | -0.1925 | No |
| 126 | DHRS7 |  |  | 7372 | -0.077 | -0.1936 | No |
| 127 | IMMT |  |  | 7374 | -0.077 | -0.1863 | No |
| 128 | G3BP2 |  |  | 7381 | -0.077 | -0.1796 | No |
| 129 | ELOVL6 |  |  | 7498 | -0.081 | -0.1852 | No |
| 130 | SSPN |  |  | 7586 | -0.085 | -0.1870 | No |
| 131 | CDKN2C |  |  | 7743 | -0.093 | -0.1961 | No |
| 132 | DHCR7 |  |  | 7806 | -0.096 | -0.1940 | No |
| 133 | ATP1B3 |  |  | 7835 | -0.098 | -0.1879 | No |
| 134 | BAZ2A |  |  | 7862 | -0.099 | -0.1813 | No |
| 135 | IFNGR1 |  |  | 7892 | -0.101 | -0.1750 | No |
| 136 | GRPEL1 |  |  | 7920 | -0.102 | -0.1683 | No |
| 137 | SPARCL1 |  |  | 8104 | -0.114 | -0.1785 | No |
| 138 | PPP1R15B |  |  | 8155 | -0.117 | -0.1729 | No |
| 139 | ATL2 |  |  | 8256 | -0.127 | -0.1723 | No |
| 140 | SLC19A1 |  |  | 8264 | -0.128 | -0.1608 | No |
| 141 | DHRS7B |  |  | 8385 | -0.144 | -0.1608 | No |
| 142 | REEP5 |  |  | 8397 | -0.146 | -0.1480 | No |
| 143 | TALDO1 |  |  | 8481 | -0.157 | -0.1424 | No |
| 144 | LAMA4 |  |  | 8483 | -0.157 | -0.1274 | No |
| 145 | CD151 |  |  | 8493 | -0.159 | -0.1131 | No |
| 146 | RTN3 |  |  | 8512 | -0.164 | -0.0994 | No |
| 147 | ITGA7 |  |  | 8599 | -0.189 | -0.0911 | No |
| 148 | LPL |  |  | 8638 | -0.208 | -0.0755 | No |
| 149 | COL4A1 |  |  | 8661 | -0.218 | -0.0571 | No |
| 150 | POR |  |  | 8682 | -0.228 | -0.0374 | No |
| 151 | ABCB8 |  |  | 8685 | -0.231 | -0.0154 | No |
| 152 | APLP2 |  |  | 8755 | -0.292 | 0.0047 | No |
Table: GSEA details [plain text format]

  

Fig 2: HALLMARK\_ADIPOGENESIS: Random ES distribution      
 Gene set null distribution of ES for **HALLMARK\_ADIPOGENESIS**
